# [Retracted] LncRNA HOTAIR controls the expression of Rab22a by sponging miR‑373 in ovarian cancer

**DOI:** 10.3892/mmr.2024.13346

**Published:** 2024-09-30

**Authors:** Zhongbao Zhang, Jiajing Cheng, Yi Wu, Jin Qiu, Yi Sun, Xiaowen Tong

Mol Med Rep 14: 2465–2472, 2016; DOI: 10.3892/mmr.2016.5572

Following the publication of this paper, it was drawn to the Editors' attention by a concerned reader that the cell apoptotic data shown in Fig. 3C and the Hoeschst 33342-stained images in Fig. 3D on p. 2468, and certain of the scratch-wound assay data shown in Fig. 5E on p. 2470 were strikingly similar to data appearing in different form in other articles written by different authors at different research institutes that had either already been published elsewhere prior to the submission of this paper to *Molecular Medicine Reports*, or were submitted for publication at around the same time. Owing to the fact that some of the abovementioned data had already apparently been published previously, the Editor of *Molecular Medicine Reports* has decided that this paper should be retracted from the Journal. The authors were asked for an explanation to account for these concerns, but the Editorial Office did not receive a reply. The Editor apologizes to the readership for any inconvenience caused.

